# CELEBRATING CENTENARIANS IN ELEMENTARY SCHOOLS: PROMOTING POSITIVE VIEWS OF AGING AMONG YOUNG CHILDREN

**DOI:** 10.1093/geroni/igad104.1131

**Published:** 2023-12-21

**Authors:** Cynthia Hancock, Tina Newsham, Daniel Alston, Elizabeth Fugate-Whitlock, Katherina Nikzad-Terhune

**Affiliations:** UNC Charlotte, Charlotte, North Carolina, United States; University of North Carolina Wilmington, Wilmington, North Carolina, United States; UNC Charlotte, Charlotte, North Carolina, United States; University of North Carolina Wilmington, Wilmington, North Carolina, United States; Northern Kentucky University, Highland Heights, Kentucky, United States

## Abstract

Upon learning that some teachers celebrate the 100th day of school by asking young children to dress up “like a 100-year-old,” the presenters questioned what ideas about aging are reinforced by such an activity. Social media images reveal that many of these activities lead to stereotypical presentations of older people. Additionally, the “fun” activity of dressing like a centenarian teaches children that it is okay to have fun at the expense of another segment of the population and reinforces the belief that older people are frail, weak, confused, and out of touch. Partnering with experts in early childhood and elementary education, the presenters created a toolkit to offer accurate information about centenarians, aging, and ageism and to give teachers options for celebrating the 100th day of school that reinforce important academic content, address prekindergarten to second-grade learning standards/outcomes, and support age-inclusivity. The presenters will share data from a pilot study of the curriculum and suggest next steps for advancing aging education among very young learners.

